# *IL7R*, *GZMA* and *CD8A* serve as potential molecular biomarkers for sepsis based on bioinformatics analysis

**DOI:** 10.3389/fimmu.2024.1445858

**Published:** 2024-11-25

**Authors:** Jin Li, Lantao Wang, Bin Yu, Jie Su, Shimin Dong

**Affiliations:** ^1^ Department of Emergency, The Fourth Hospital of Hebei Medical University, Shijiazhuang, Hebei, China; ^2^ Department of Emergency, Third Hospital of Hebei Medical University, Shijiazhuang, Hebei, China

**Keywords:** bioinformatics analysis, CD8A, emergency, GZMA, IL7R, sepsis

## Abstract

**Purpose:**

Sepsis is an unusual systemic reaction to what is sometimes an otherwise ordinary infection, and it probably represents a pattern of response by the immune system to injury. However, the relationship between biomarkers and sepsis remains unclear. This study aimed to find potential molecular biomarkers, which could do some help to patients with sepsis.

**Methods:**

The sepsis dataset GSE28750, GSE57065 was downloaded from the GEO database, and ten patients with or without sepsis from our hospital were admitted for RNA-seq and the differentially expressed genes (DEGs) were screened. The Metascape database was used for functional enrichment analysis and was used to found the differential gene list. Protein-protein interaction network was used and further analyzed by using Cytoscape and STRING. Logistic regression and Correlation analysis were used to find the potential molecular biomarkers.

**Results:**

Taking the intersection of the three datasets yielded 287 differential genes. The enrichment results included Neutrophil degranulation, leukocyte activation, immune effectors process, positive regulation of immune response, regulation of leukocyte activation. The top 10 key genes of PPI connectivity were screened using cytoHubba plugin, which were *KLRK1*, *KLRB1*, *IL7R*, *GZMA*, *CD27*, *PRF1*, *CD8A*, *CD2*, *IL2RB*, and *GZMB*. All of the hub genes are higher expressed in health group of different databases. Logistic regression showed that *IL7R*, *GZMA* and *CD8A* proteins were analyzed and all of them were statistically significant. Correlation analysis showed that there was a statistically significant correlation between *IL7R*, *GZMA* and *CD8A*.

**Conclusion:**

*KLRK1*, *KLRB1*, *IL7R*, *GZMA*, *CD27*, *PRF1*, *CD8A*, *CD2*, *IL2RB*, *GZMB* are key genes in sepsis, which associated with the development of sepsis. However, *IL7R*, *GZMA* and *CD8A* may serve as the attractively potential molecular biomarkers for sepsis.

## Introduction

Sepsis is characterized by a dysregulated host immune response to infection (1). Sepsis-induced immunosuppression is resulted from disruption of immune homeostasis. It is characterized by the release of anti-inflammatory cytokines, abnormal death of immune effector cells, hyperproliferation of immune suppressor cells, and expression of immune checkpoints (2).

With the efforts of critical care specialists around the world applying anti-infective combined with fluid resuscitation and organ function support, the mortality rate of septic patients is still as high as 20%, although it has been significantly reduced (3). The pathophysiologic mechanisms of sepsis are complex, and relevant indicators are not easily accessible, making early diagnosis difficult in the clinic. Therefore, understanding the pathophysiological mechanisms of sepsis pathogenesis and identifying potential diagnostic and therapeutic biomarkers are essential for improving clinical outcomes and reducing mortality in sepsis patients (4–6). However, the relationship between biomarkers and sepsis remains unclear.

Bioinformatics was used to analyze genomic and proteomic data, which may do some help to predicting novel genes of potential value accurately (7). In recent years, bioinformatics analysis has been widely used for the detection and analysis of differential gene expression in sepsis (8–11). Currently, there is little exploration of the role of inflammatory factors in sepsis through sequencing and big data analysis. Therefore, it is necessary to explore the role of inflammatory factors in sepsis through our clinical sample sequencing combined with database data. This study aimed to find potential molecular biomarkers, which could do some help to patients with sepsis.

## Materials and methods

### Subject recruitment and blood collection

In the present study, we analyzed 6 patients with sepsis who were admitted to the emergency intensive care unit of the Fourth Hospital of Hebei Medical University. The recruitment lasted from May 2023 to July 2023. Their blood samples were collected within 24 hours after the admission and were analyzed. Peripheral blood samples were also collected from the control group, they were 4 healthy individuals. Inclusion criteria were as followed: patients were diagnosed with sepsis and were admitted to emergency intensive care unit, sepsis diagnosed with sepsis 3.0 definition and diagnosis standard (infection + ΔSOFA scoring ≥2) jointly issued by the Society of Critical Care Medicine and European Society of Intensive Care Medicine, age ≥18 and ≤65 years, and subjects or legal representatives willing to participate in the study and to sign an informed consent form. Exclusion criteria were as followed: those with previous organ failure(Including heart valve disease, nephritis, nephrotic syndrome, etc), those with previous immune system diseases, those with previous blood system diseases, patients unwilling to participate. Our study plans were approved by the Ethics Committee of our hospital.

### RNA-seq

In the first 24 hours after the patients’ admission, their blood samples were collected with the PAXgene Blood RNA Tube (PreAnalytix GmbH, Switzerland)and were stored in the refrigerator in our hospital until further analysis. After extraction of RNA samples, quality control detection was made with Bioanalyzer 2100 (Agilent, CA, USA), and mRNA was required to meet Concentration >50ng/μL, RIN value >7.0, total RNA >1μg to satisfy downstream experiments. After sample quality control was passed, a DNA database was built, and RNA-seq was made with Illumina NovaseqTM 6000.

### Sepsis dataset

In the present study, the GSE28750, GSE57065 dataset was downloaded from the GEO online database (www.ncbi.nlm.nih.gov/geo). In our study, 10 peripheral blood samples of sepsis patients were selected from GSE28750 as the sepsis group, and 20 peripheral blood samples of healthy volunteers as the control group; 28 peripheral blood samples of sepsis patients were selected from GSE57065 as the sepsis group, and 25 peripheral blood samples of healthy volunteers as the control group.

### Screening of differential genes

Differential expression genes (DEGs) between the healthy group and the sepsis group were screened using DESeq2. The R package limma (version 3.40.6) was used to explore the DEGs between sepsis and non-sepsis peripheral blood. The result of RNA-seq (sepsis patients VS. non-sepsis participants) dataset was analyzed using the lmFit method for multivariate regression. The Bayes method was used to calculate the moderated t-statistics, moderated f-statistics and log ratios of differential expression by empirical Bayesian adjustment. A multiplicity of differences |FC|≥1.5 and a *q*-value of <0.05 was used as the threshold criterion (no multiplicity of differences in the multiple group comparisons, and genes screened for *q*<0.05 were genes statistically different between the multiple groups). Volcano plots were used to find the DEGs.

### Functional enrichment analysis

The Metascape database (https://metascape.org/gp/index.html#/main/step1) provides a list of annotations and resources and can visually export them. The Metascape database was used for functional enrichment analysis and was used to found the differential gene list.

### Protein-protein interaction analysis

Protein-protein interaction (PPI) network is used to screen potential core genes. In our study, STRING (https://string-db.org/) online platform was used to screen core genes of DEGs. By using the multi-protein online tool in the STRING database, the PPI networks were predicted. The results were showed with Cytoscape 3.6.1The most critical modules were then found using the Complex Detection (MCODE) plugin.

### Statistical analysis

The data were analyzed by using Graph PadPrism version 9.0 (GraphPad Software, Inc.). For comparisons between two groups, an unpaired, 2-tailed Student’s t-test was used. The data is presented as the mean ± standard deviation. Univariate and multivariate logistic regression analyses were performed to analyze the potential molecular biomarkers. *P*<0.05 was considered to indicate a statistically significant difference.

## Results

### Differentially expressed genes

**Figure 1:**
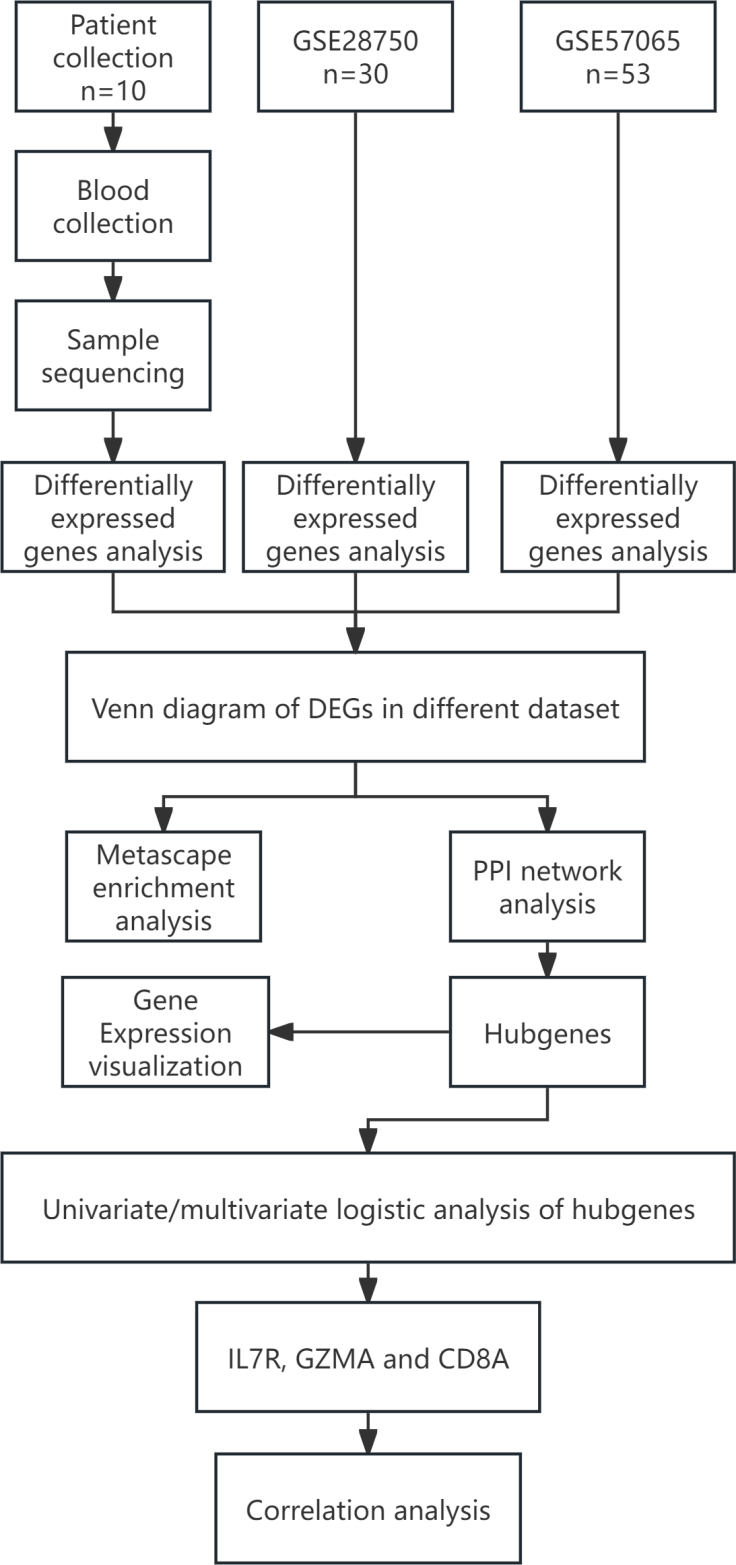
Flow chart of the statistical analysis. DEGs, Differentially Expressed Genes; PPI, protein-protein interaction.st.

**Figure 2:**
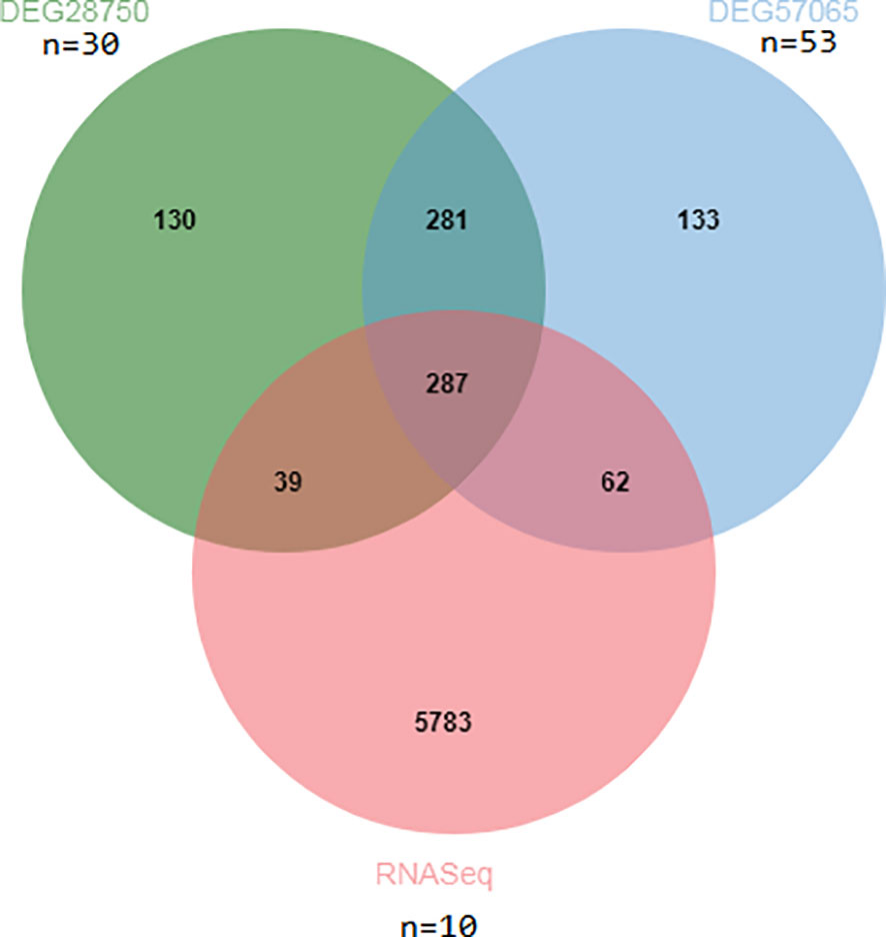
Venn diagram of DEGs in different dataset.

**Figure 3:**
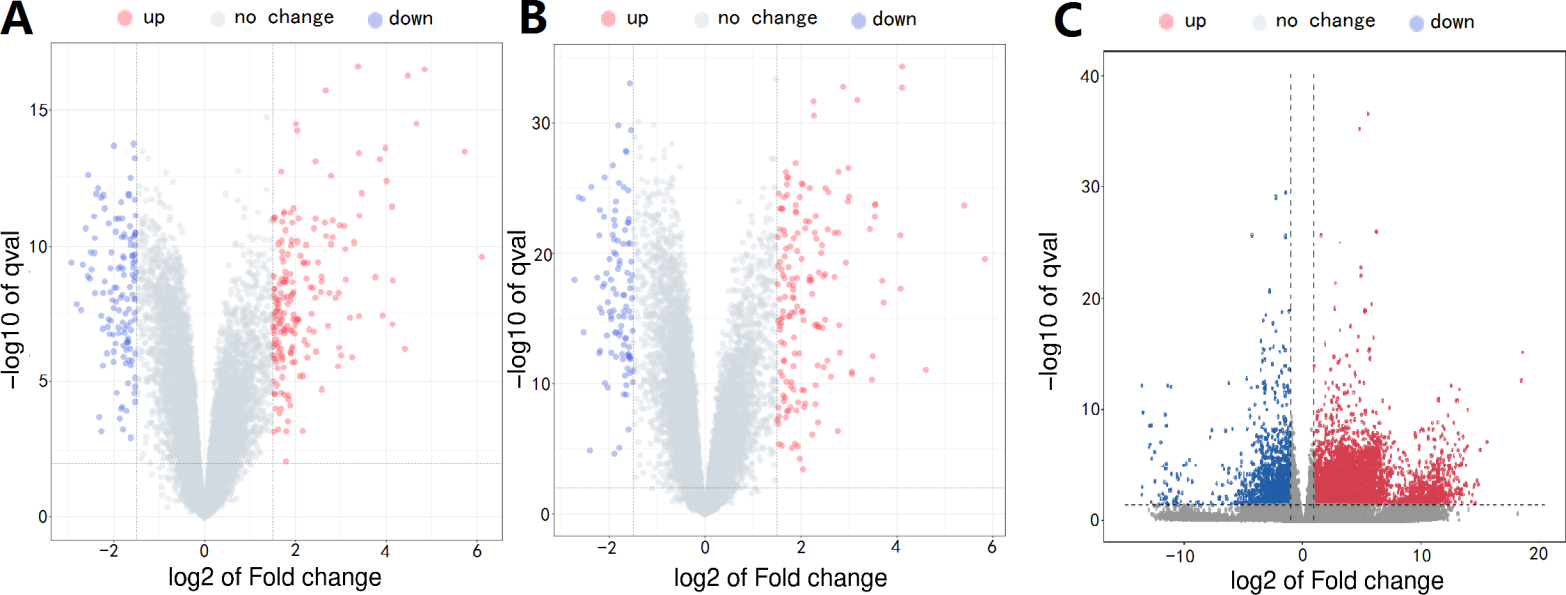
**(A)** GSE28750 microarray, **(B)** GSE57065 microarray, **(C)** RNA-seq, Red, relatively high expression; Blue, relatively low expression.

The design concept of the article was represented by a flowchart (**Figure 1**). 244 differential genes were screened from GSE28750, including 152 expression up-regulated genes and 92 expression down-regulated genes, and 264 differential genes were screened from GSE57065, including 163 expression up-regulated genes and 103 expression down-regulated genes. A total of 6,171 differentially expressed genes were obtained between sepsis and normal control groups, including 4625 up-regulated and 1546 down-regulated expressed genes. Taking the intersection of the three datasets yielded 287 differential genes (**Figure 2**). Three volcano plots showed up- and down-regulated expression genes (**Figure 3**).

### Metascape enrichment analysis

**Figure 4:**
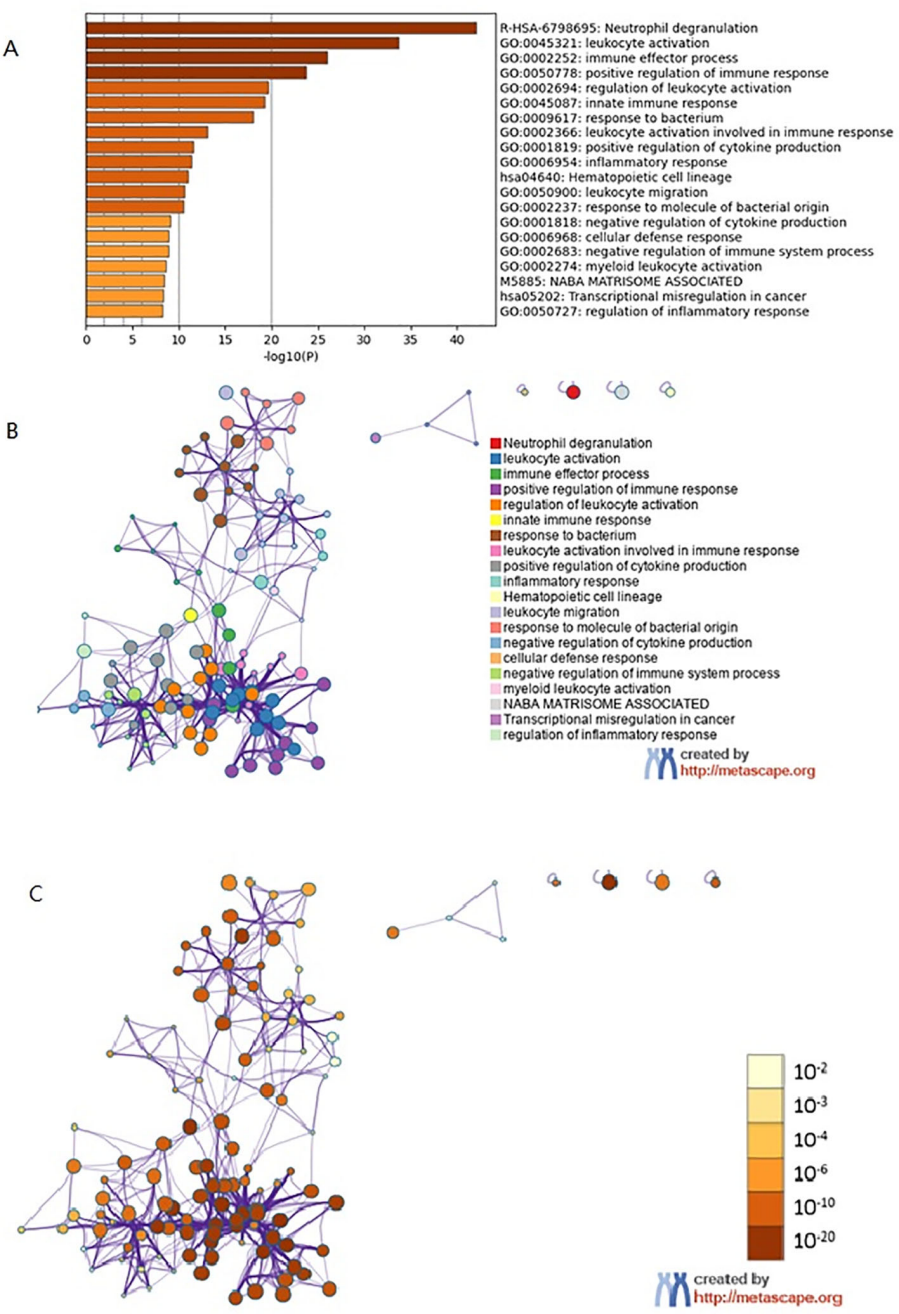
Metascape enrichment analysis. **(A)** GO enrichment terms. **(B)** Enrichment network colored by enrichment terms. **(C)** Enrichment network colored by *p*-value.

**Figure 5:**
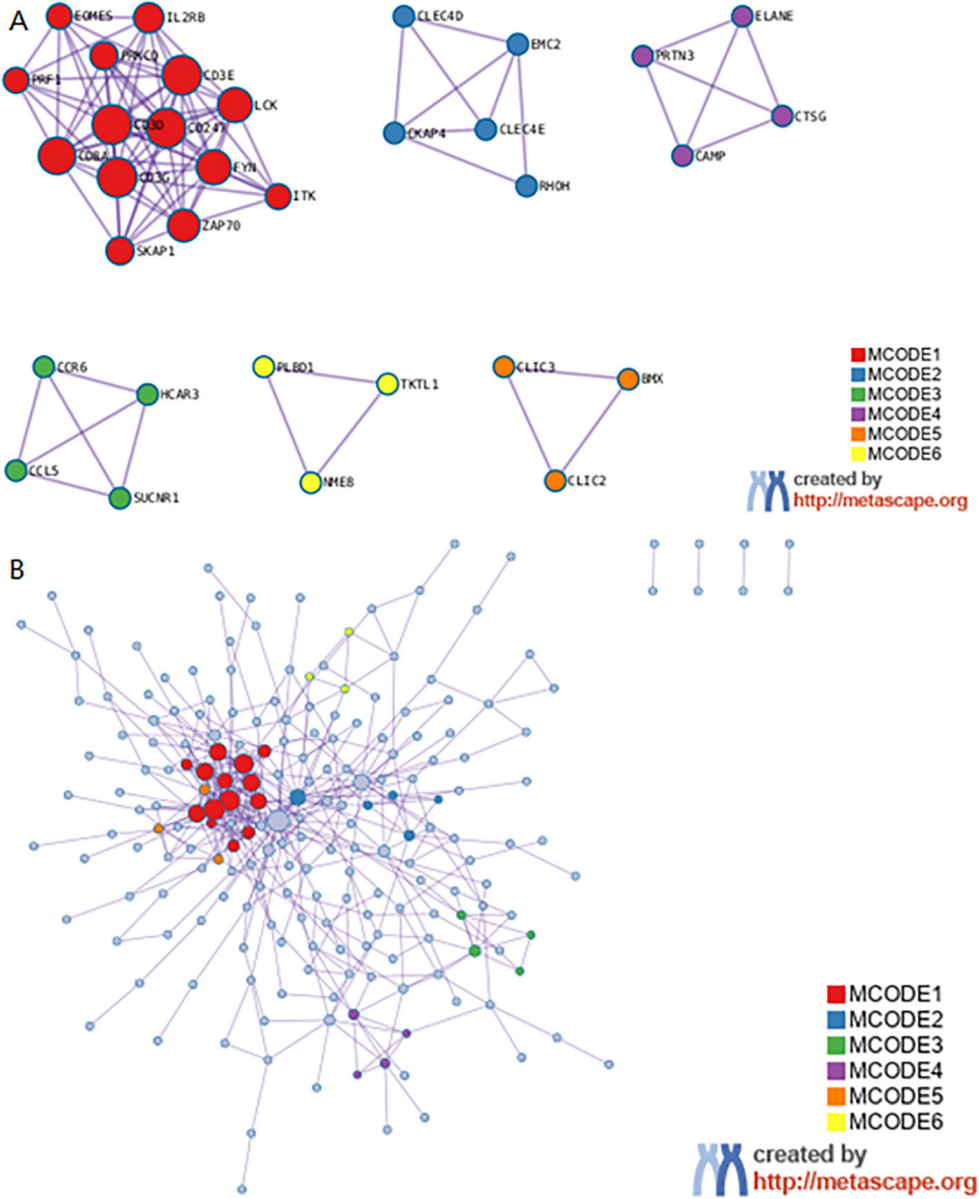
Metascape enrichment analysis. **(A)** The significant model. **(B)** The protein-protein interaction network.

Metascape enrichment shows GO enrichment terms (**Figure 4A**) and enrichment networks colored with enrichment terms and *p*-values (**Figures 4B, C**, **5**). **Figure 4A** is a bar graph of enriched terms across input gene lists, colored by *p*-values. The enrichment results included Neutrophil degranulation, leukocyte activation, immune effector process, positive regulation of immune response, regulation of leukocyte activation. **Figure 4B** shows the network of enriched terms. The enriched results were colored with cluster ID, where nodes sharing the same cluster ID are typically close to each other. The **Figure 4C** shows the enrichment result items colored with *P*-values. Items with more genes in them tend to have more significant *P*-values. As shown in the **Figure 5**, the significant models of genes were shown in **Figure 5A**, which was analyzed by MCODE. The significant models of genes were also shown in the whole Protein-protein interaction network.

### Protein-protein interaction network analysis and identification of hub genes

**Figure 6:**
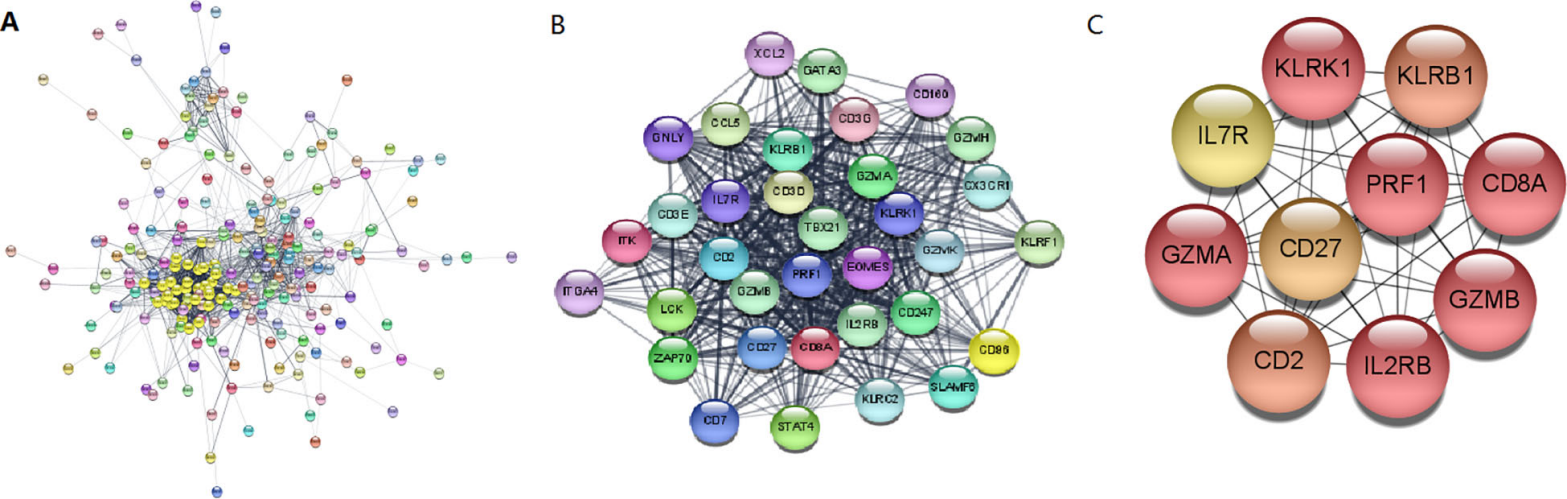
Construction and analysis of the PPI networks. **(A)** PPI network of DEGs. **(B)** The interaction of core genes. **(C)** The top 10 hub genes. hub genes: *KLRK1*, *KLRB1*, *IL7R*, *GZMA*, *CD27*, *PRF1*, *CD8A*, *CD2*, *IL2RB*, *GZMB*. PPI, protein-protein interaction; DEG, differentially expressed gene.

The Search Tool for Retrieval of 31 Interacting EP-DEG online database was used to create protein network interaction maps. By using the multi-protein online tool in the STRING database, the PPI networks were predicted. The results were showed with Cytoscape 3.6.1. The most critical modules were then found using the Complex Detection (MCODE) plugin. The PPI network obtained from the visual analysis of Cytoscape software included 281 nodes and 1639 edges, **Figure 6A**; the most significant modules were found using the MCODE plug-in, which contained a total of 34 genes, see **Figure 6B**. The top 10 key genes of PPI connectivity were screened using cytoHubba plugin, which were *KLRK1*, *KLRB1*, *IL7R*, *GZMA*, *CD27*, *PRF1*, *CD8A*, *CD2*, *IL2RB*, *GZMB* (**Figure 6C**).

### Expression of key genes in sepsis and healthy groups

**Figure 7:**
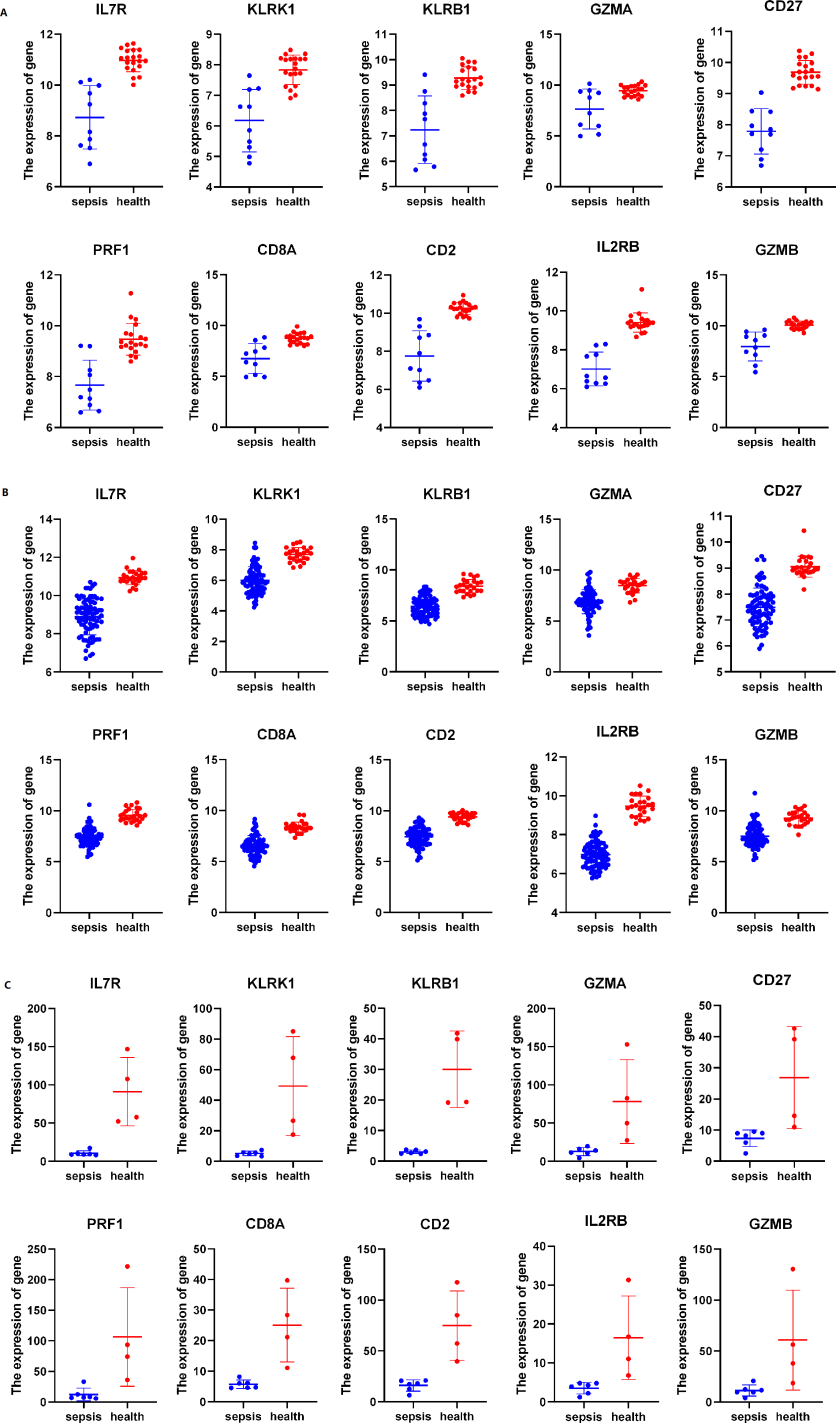
The expression of hub genes in different dataset **(A)** The expression of genes in GSE28750, **(B)** The expression of genes in GSE57065, **(C)** RNA seq of our dataset. All data was shown by mean ± SD.

All of the hub genes (*IL7R*, *KLRK1*, *KLRB1*, *GZMA*, *CD27*, *PRF1*, *CD8A*, *CD2*, *IL2RB*, *GZMB*) are higher expressed in health group of different databases (**Figures 7A–C**).

### Univariate and multivariate logistic regression analyses of hubgenes

**Figure 8:**
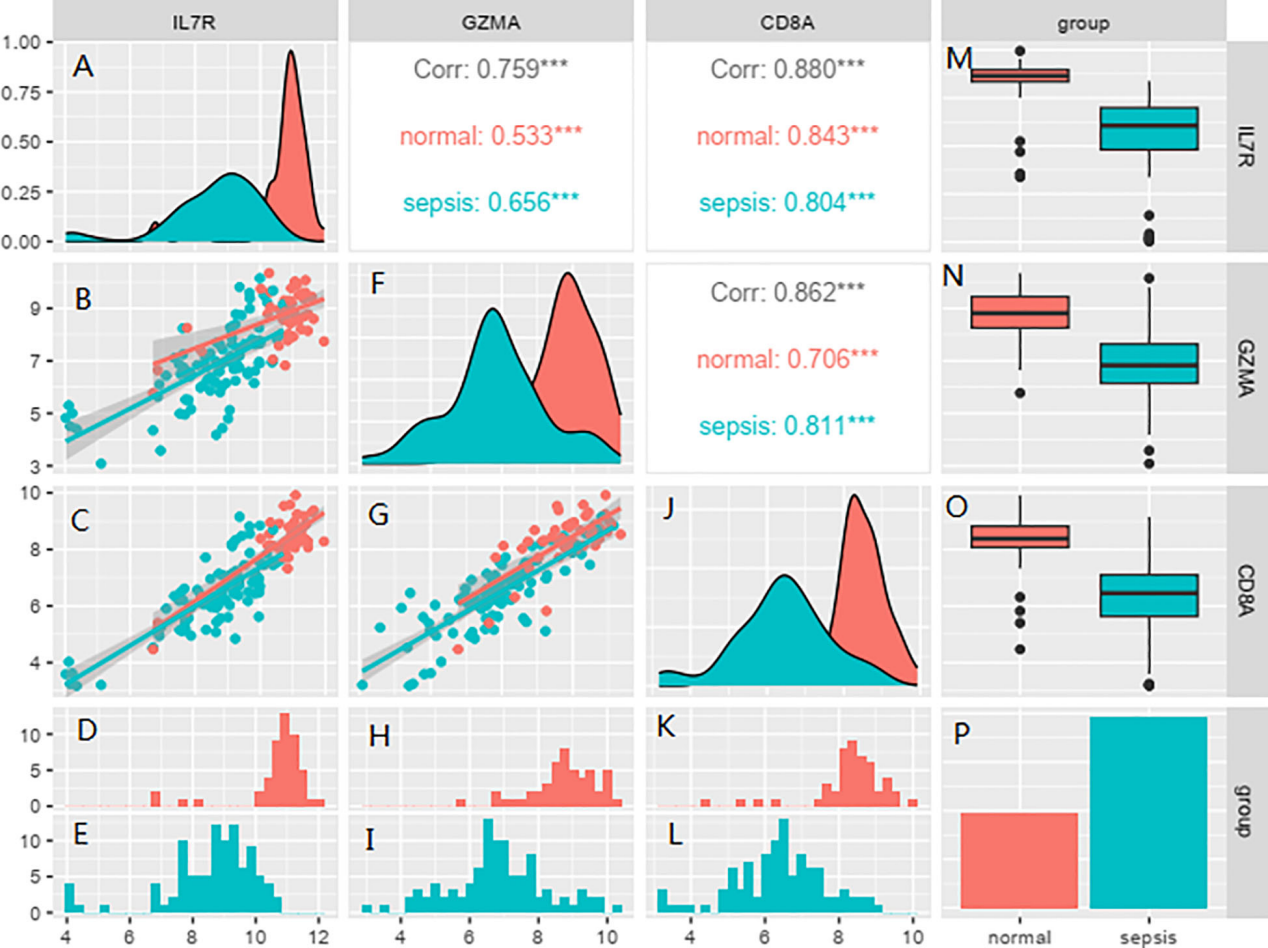
Correlation analysis showed that there was a correlation between the *IL7R*, *GZMA* and *CD8A*. Red represents normal subjects, and blue represents sepsis patients. **(A, F, J)** are the abundance maps of gene expression in different groups, **(B, C, G)** are the scatter plots of correlation analysis of corresponding gene expression in different groups, **(D, H, K)** are the gene expression levels of each patient in the normal group, **(E, I, L)** are the expression levels of corresponding genes in each patient in the sepsis group, **(M, N, O)** are the line box plots of corresponding gene expression in different groups, presented in the form of mean ± standard deviation. **(P)** shows the sepsis group and the healthy group.

In this study we applied logistic regression for analysis and the results showed that *IL7R*, *GZMA* and *CD8A* proteins were analyzed and all of them were statistically significant, so we analyzed these 3 proteins for subsequent experiments (**Tables 1**, **2**). Correlation analysis showed that there was a correlation between the two *IL7R*, *GZMA* and *CD8A* proteins and the correlation was statistically significant (**Figure 8**).

**Table 1:** Univariate analysis of hub-genes in sepsis.

Genes	*P*	HR	95%CI	
			LowerCI	UpperCI
IL7R	<0.05*	6.498	3.539	11.933
KLRK1	<0.05*	9.058	4.467	18.366
KLRB1	<0.05*	5.304	3.136	8.973
GZMA	<0.05*	3.534	2.341	5.333
CD27	<0.05*	5.566	3.207	9.661
PRF1	<0.05*	9.468	4.641	19.316
CD8A	<0.05*	4.728	2.863	7.806
CD2	<0.05*	8.43	4.298	16.534
IL2RB	<0.05*	5.704	3.407	9.551
GZMB	<0.05*	3.925	2.53	6.088

* *P*<0.05.

**Table 2:** Multivariate analysis of hub-genes in sepsis.

Genes	*P*	HR	EXP(B)95%CI	
			LowerCI	UpperCI
IL7R	0.045*	0.015	0	0.911
KLRC4-KLRK1	0.044*	1946.567	1.246	3042206
KLRB1	0.129	21.489	0.409	1129.43
GZMA	0.046*	0.003	0	0.891
CD27	0.464	0.319	0.015	6.826
PRF1	0.052	254.578	0.944	68653.94
CD8A	0.012*	0	0	0.147
CD2	0.003*	272452.6	62.228	1.19E+09
IL2RB	0.58	3.354	0.046	244.811
GZMB	0.113	0.039	0.001	2.142

* *P*<0.05.

## Discussion

Sepsis is described as the syndrome consisting of complex biochemical and pathophysiological dysregulation (12). Dysregulation of the body’s inflammatory response followed by the promotion of an inflammatory cascade is an important basis for the pathophysiologic changes in sepsis (13). Multiple pathophysiologic processes are involved in septic organ damage, and immunoregulatory imbalance is one of the important mechanisms (14). At the cellular and molecular level, the mechanisms include dysregulation of the inflammatory response, immunosuppression, coagulation disorders, apoptosis of immune cells, endoplasmic reticulum stress, and other pathophysiological processes (15).

Mechanisms of sepsis have been reported by investigators, but few of them have addressed its molecular markers; in fact, molecular biomarkers have great potential for the diagnosis, monitoring and prognosis of sepsis. Using the sequencing technology, the use of RNA-seq in subbiology has become very common, and its role has expanded the understanding of tiny molecules (16, 17).

Taking the intersection of the three datasets, we find 287 DEGs. Moreover, The enrichment results included Neutrophil degranulation, leukocyte activation, immune effector process, positive regulation of immune response, regulation of leukocyte activation. It has been shown that DEGs are associated with the process of apoptosis and immune system, further, studies have confirmed that apoptosis of immune cells promotes the process of organ dysfunction along with immune failure in patients with sepsis (18). Innate immune cells recognize pathogens and release pro-inflammatory cytokines such as *TNF-α*, *IL-1*, *IL-6*, which play an immune defense role; at the same time, they also negatively elevate anti-inflammatory cytokines such as *TGF-β*, *IL-4*, and *IL-10 (* 19–21). It is now believed that sepsis is caused by excessive systemic inflammation leading to immune dysfunction, and that immune cells such as neutrophils, macrophages, and T-lymphocytes are involved in the regulation of the inflammatory response (22). By using cytoHubba plugin, the top 10 genes *KLRK1*, *KLRB1*, *IL7R*, *GZMA*, *CD27*, *PRF1*, *CD8A*, *CD2*, *IL2RB*, and *GZMB* were found and more of these hub-genes have high relationship with immune system. Therefore, keeping an eye on the expression of these genes in the blood can help clinicians make clinical decisions that will benefit their patients. After initially analyzing the more important 10 hub-genes, we further performed Univariate and multivariate logistic regression based on the patients’ clinical information, and found that three genes, *IL7R*, *GZMA* and *CD8A*, were statistically significant in the multivariate logistic regression, which implies that these three genes are independent risk factors for determining sepsis. This provides clinicians with a basis for decision-making in the diagnosis, treatment, and prediction of clinical prognosis for patients with sepsis.

Imbalance of the immune response is one of the main mechanisms of sepsis, and immune dysfunction in sepsis is closely related to T lymphocytes (23). *CD4*, *CD8A*, *CD28*, *CD2*, *CD3E* as T cell surface active molecules play important roles in the immune response process; among them, the roles of *CD4* and *CD8* T lymphocytes in sepsis have been widely recognized (24). Several previous studies have demonstrated the *CD28*, *CD3*, *CD4* molecules in sepsis or septic shock (25, 26). Study showed some hub genes (*CD2*, *CD27*, *GZMA*, *KLRB1*, and *PRF1*) have been screened out as sepsis biomarkers and all of them were down regulated genes in sepsis, which consistent with our findings (27).

The protein encoded by *CD2* is a surface antigen found on all peripheral blood T cells. A study also found that *CD2* is identified as the down regulated crucial gene set in sepsis (28). The protein encoded by *CD27* is a member of the tumor necrosis factor receptor superfamily and is also necessary for the production and long-term maintenance of T cell immunity. *CD27* may help identify preterm infants with sepsis and may also help clinicians identify children at high risk (29). *CD8A* encodes the cd8α chain of the dimeric *CD8* protein. *CD8A* is primarily involved in cell-mediated immune defense and T cell development (30, 31). *CD8* deficiency increases susceptibility to infection (32). Harland et al (24) revealed that cpG methylation of the cd8a locus has a potential role in the downregulation of *CD8*.

*IL7*, an important member of the chemokine family, mediates the immune response by binding to the receptor *IL7R* to promote lymphocyte growth, macrophage activation, and cytokine and inflammatory factor secretion (33). Some studies had shown that the level of *IL7R* in sepsis was low expressed, which was consistent with our findings (34). Animal experiments have shown that activation of the *IL-7*/*IL-7R* signaling pathway improves survival in septic mice (35). Some clinical studies have also suggested that the *IL-7*/*IL-7R* signaling pathway is associated with improved lymphocyte function in sepsis patients (36). *IL2RB* is a subunit of *IL2R* and is closely related to humoral immunity as it triggers *IL2R* through *IL2* binding, leading to proliferation and differentiation of a large number of immune cells, including T cells, B cells and macrophages. *IL2RB* is recognized as an indicator of sequential organ failure and is negatively correlated with sepsis mortality (37). Another study showed that targeting *IL2RB* can do some help to reduce acute lung injury caused by sepsis (38).

*PRF1* is a perforin protein secreted by natural killer cells, cytotoxic T-lymphocytes and T-cells, which plays an important role in immunoregulation and immunosurveillance, and has been widely studied in tumors and immune diseases (39). Some studies have found that *PRF1* gene expression is reduced in T cells and natural killer cells in patients with sepsis, but the exact mechanism of action still needs to be further clarified (40). *GZMA* is a serine protease specific for T cells and NK cells. It may be a common component for cytotoxic T lymphocytes and NK cells to cleave target cells. Study showed that *GZMA* was a key gene of the inflammatory response during abdominal sepsis (41). A study also showed the inhibition of *GZMA* can reduce inflammation and improve survival during Escherichia coli sepsis (42). *GZMB* is a member of grazymes family that was considered to exert cytotoxic effects against pathogen invasion (43). Some studies reported that *GZMB* is involved in the coagulation cascade, regulating the function of platelets and endothelial barrier permeability in sepsis (44). Study showed *GZMB* have potential diagnostic value in sepsis diagnosis (45). *KLRK1* is known as homo sapiens killer cell lectin-like receptor subfamily K, member 1. Viral or bacterial infection can lead to the induction of *KLRK1* ligands on cells, which can activate the immune system to recognize and eliminate them (46). *KLRB1*, known as killer cell lectin like receptor B1, is a gene encoding *CD161*. *CD161* is expressed on immune cells (47). Study showed that *KLRB1* was identified as the downregulated crucial gene set in sepsis (28).

The stability of mRNA is influenced by temperature, mRNA length, concentration, pH value, and buffer type, which in turn affects protein expression (48). A study shows that attenuating ribosome load improves protein output from mRNA by limiting translation-dependent mRNA decay (49). Another study states that, cytidine-containing tails robustly enhance and prolong protein production of synthetic mRNA in cell and *in vivo (* 50). Although the half-life of mRNA has a significant impact on protein formation, it does not affect the conclusion of the data analysis in this article that IL7R, GZMA and CD8A may serve as the attractively potential molecular biomarkers for sepsis.

## Conclusion

Based on the discussion and analysis, it is reasonable to assume that *IL7R*, *GZMA* and *CD8A* possess significant potential value in the diagnosis and prediction of sepsis. We have found that the immune system plays an important role in the development of sepsis, so intervening or modulating the balance of the immune system in the body may benefit sepsis patients in clinical diagnosis and treatment. Our study also had the limitation of a small sample size, so we used correlation analysis to identify key genes for further clinical trial validation.

## Data Availability

The datasets presented in this study can be found in online repositories. The names of the repository/repositories and accession number(s) can be found in the article/supplementary material.
